# Managing boundaries in primary care service improvement: A developmental approach to communities of practice

**DOI:** 10.1186/1748-5908-7-97

**Published:** 2012-10-15

**Authors:** Roman Kislov, Kieran Walshe, Gill Harvey

**Affiliations:** 1 Manchester Business School, The University of Manchester, Booth Street West, Manchester, M15 6PB, UK

**Keywords:** Communities of practice, Professional boundaries, Organisational boundaries, Primary care, Service improvement, National Health Service, CLAHRC, UK

## Abstract

**Background:**

Effective implementation of change in healthcare organisations involves multiple professional and organisational groups and is often impeded by professional and organisational boundaries that present relatively impermeable barriers to sharing knowledge and spreading work practices. Informed by the theory of communities of practice (CoPs), this study explored the effects of intra-organisational and inter-organisational boundaries on the implementation of service improvement within and across primary healthcare settings and on the development of multiprofessional and multi-organisational CoPs during this process.

**Methods:**

The study was conducted within the Collaboration for Leadership in Applied Health Research and Care (CLAHRC) for Greater Manchester—a collaborative partnership between the University of Manchester and local National Health Service organisations aiming to undertake applied health research and enhance its implementation in clinical practice. It deployed a qualitative embedded case study design, encompassing semistructured interviews, direct observation and documentary analysis, conducted in 2010–2011. The sample included practice doctors, nurses, managers and members of the CLAHRC implementation team.

**Findings:**

The study showed that in spite of epistemic and status differences, professional boundaries between general practitioners, practice nurses and practice managers co-located in the same practice over a relatively long period of time could be successfully bridged, leading to the formation of multiprofessional CoPs. While knowledge circulated relatively easily within these CoPs, barriers to knowledge sharing emerged at the boundary separating them from other groups existing in the same primary care setting. The strongest boundaries, however, lay between individual general practices, with inter-organisational knowledge sharing and collaboration between them remaining unequally developed across different areas due to historical factors, competition and strong organisational identification. Manipulated emergence of multi-organisational CoPs in the context of primary care may thus be problematic.

**Conclusions:**

In cases when manipulated emergence of new CoPs is problematic, boundary issues could be addressed by adopting a developmental perspective on CoPs, which provides an alternative to the analytical and instrumental perspectives previously described in the CoP literature. This perspective implies a pragmatic, situational approach to mapping existing CoPs and their characteristics and potentially modifying them in the process of service improvement through the combination of internal and external facilitation.

## Background

Effective implementation of change in healthcare organisations involves multiple professional and organisational groups and is often impeded by professional and organisational boundaries that present relatively impermeable barriers to sharing knowledge and spreading work practices [1]. Professional boundaries in healthcare are reinforced by historically determined power and status differentials between healthcare professionals [2] whereas organisational boundaries could be caused by the government’s imposition of divergent performance frameworks upon organisations that are expected to collaborate, with explicit incentives for collaboration frequently being absent [3]. A complementary view of professional and organisational barriers to knowledge sharing is suggested by practice-based theorists who maintain that knowledge is localised, embedded and invested in collective practice and see boundaries as inherent sociocultural differences between distinct collective practices underpinned by shared language, meanings and ways of doing things [4-6]. According to this tradition, the effect of these differences is dual: they can lead to innovation, learning and cross-fertilisation between practices, on the one hand, and to separation, fragmentation and disconnection, on the other [7,8].

One of the practice-based theories specifically exploring boundaries between different sets of practice is the *communities of practice* (CoP) approach developed by Jean Lave and Etienne Wenger [9,10] and applied to the analysis of learning, practice, meaning and identity in various contexts, including organisational studies [4] and healthcare [11,12]. CoPs are work-related communities of individuals created over time through sustained collective pursuits of shared enterprises [4,10,11]. Shared knowledge, practice and identity produce boundaries between CoPs, at which they differentiate themselves from and also interlock with other communities, forming complex social landscapes of practice. An inherent feature of the CoP landscape is actors’ multimembership in various CoPs which can be crucial for bridging boundaries between communities [10,13]. It should be noted that CoP boundaries do not usually coincide with the organisational ones. On the one hand, organisations represent a multiplicity of subcultures and could at best be seen as ‘constellations of interconnected practices’ [10] or ‘communities-of-communities’ [4]. On the other hand, some of the CoPs may cut across organisational boundaries, with their members potentially transferring knowledge between organisations [5,14].

At the same time, CoP boundaries are often seen as a reproduction of professional boundaries, with the possibility of multiprofessional CoP formation in healthcare being contested. For example, in their study of eight National Health Service (NHS) innovations, Ferlie et al. [11] show that CoPs in healthcare are predominantly uniprofessional, tend to seal themselves off from neighbouring professional communities and are highly institutionalised, which enables a relatively easy flow of knowledge within these CoPs but causes the ‘stickiness’ of knowledge across boundaries and hence retards the innovation spread. They argue that great effort is needed to bridge professional boundaries and create a functioning multiprofessional CoP because uniprofessional CoPs tend to defend their jurisdictions and group identity. The construction of such a CoP was observed by Ferlie and colleagues only in one of their primary care cases, where professional boundaries could be successfully bridged because GPs and practice nurses shared common values; participation in change was incentivised; established systems for interprofessional dialogue were deployed; and basic cognitive assumptions of professional groups remained unchallenged.

A more optimistic view on multiprofessional CoPs is presented by Gabbay and le May [15] in an ethnographic account of knowledge sharing in primary care, which describes a system of overlapping ‘communities of general practice’ existing in primary care organisations. In addition to the uniprofessional ‘coffee room GPs’ community which is at the centre of their analysis, the authors also identify several ‘specialist’ CoPs located within the same practice, some of which are multiprofessional; wider (and looser) CoPs external to the practice (e.g. a group of fellow managers for the practice manager or a network of old colleagues for practice doctors); and, interestingly, a multiprofessional CoP that evolved from formal practice meetings and included almost all of the staff in the practice. Another example of a multiprofessional CoP operating in primary care is provided by Hudson [16], who describes a multi-agency team working at the interface of district nursing and social work and argues that the promotion of shared values and socialisation to an immediate work group can override professional or hierarchical differences amongst staff and lead to the formation of a multiprofessional and multi-organisational CoP.

Empirical studies outlined above deployed the *analytical perspective* on CoPs, whereby this theory is applied to the analysis of processes that take place in organic CoPs naturally emerging as a product of collective practice over a relatively long period of time. Another strand of CoP informed thinking, which has been labelled as the *instrumental perspective*[13,17] is concerned with the deliberate cultivation of CoPs in order to bridge professional and organisational boundaries and enable knowledge transfer [18]. Deliberately constructed CoPs have been shown to be effective in enhancing professional education, adoption of innovation and problem-solving [19,20]. In service improvement, CoP cultivation has been specifically advocated as an approach useful for creating horizontal networks across organisations, promoting the sharing of tacit knowledge and achieving a better sustainability of change [21,22]. However, emergence of genuine CoPs within quality improvement and other change implementation initiatives in the healthcare sector may be problematic due to the time-limited nature of the projects, a top-down approach to change management and preoccupation with performance measurement at the expense of human and social aspects of change [3,22,23]. In addition, administrative staff, nurses, medical practitioners, allied health professionals and managers have been shown to significantly differ in their conceptualisations of quality and safety, which may challenge the collaborative implementation of service improvement initiatives in a multiprofessional environment [24,25]. Interestingly, while the instrumental perspective on CoPs sees bridging the boundaries as its target, the impact of pre-existing professional and organisational boundaries on CoP engineering in healthcare collaboration seems somewhat underestimated in the literature [17].

The brief outline of literature presented above points to a number of aspects requiring more empirical attention. First, boundaries to knowledge sharing in healthcare have been predominantly explored in secondary care settings [26,27], at the interface between the primary and secondary care sectors [28,29], and in partnerships between NHS organisations and higher medical education [3]. At the same time, the nature and effects of boundaries existing in the primary healthcare sector may be different from those found in secondary care and intersectoral collaboration. Little is known about how complex landscapes of practice comprised of multiple CoPs influence intra- and inter-organisational knowledge sharing, especially in the process of service improvement initiatives introduced in primary care. Second, while there is a growing number of studies exploring the deliberate cultivation of CoPs in order to promote evidence-based practice, foster collaboration and achieve service improvement in healthcare, less is known about how professional and organisational boundaries shape the development of new CoPs within these initiatives, whether the manipulated emergence of new multiprofessional and multi-organisational CoPs in primary care is realistic, and how these groups relate to pre-existing organic CoPs.

In light of the above, this study is guided by the following research questions:

1. How do boundaries between CoPs existing within and across general practices influence the implementation of a primary care service improvement programme?

2. How do these boundaries affect the emergence of new multiprofessional and multi-organisational CoPs within and across primary care organisations?

Before discussing the methodology deployed to address these research questions, it is worth clarifying the definitions of boundaries and communities of practice used in this study. In line with the practice-based approach to knowledge sharing, we define boundaries as sociocultural differences between groups that may lead to discontinuity in action or interaction [7]. Our understanding of boundaries, therefore, partially overlaps with the notion of ‘gaps’ popular in the knowledge transfer literature, where gaps are seen as ‘the network holes, spaces and missing ties that create between-group problems and opportunities for their resolution’ [30]. It could, however, be argued that the latter approach emphasises structural and relational separation between groups that can be ‘bridged’ by ‘transferring’ knowledge from one group to another through routines, protocols and other information channels. By contrast, our understanding of boundaries as discontinuities, underpinned by differences between groups in terms of practices, identities and meanings, highlights the cultural and political nature of these phenomena, shifts the focus of analysis from ‘gaps’ and ‘bridges’ to divergent meanings, interests and cultures, and underscores the importance of reflection, learning and transformation when dealing with boundaries [31,32].

Following Wenger’s seminal analytical text on CoPs [10], we define a CoP as a group of individuals created over time through sustained collective pursuit of a joint enterprise and developing mutual engagement with each other as well as a shared repertoire of meanings, routines, stories and artefacts. In line with Wenger’s theory, CoPs are also characterised by the presence of boundaries, shared identities and collective histories of learning. It should be noted, however, that we do not accept the clearcut and sometimes criticised [17,33] dichotomy between teams and CoPs postulated in Wenger’s later writings [18,34]. Based on our own previous empirical research [35], we argue that some teams can develop certain CoP characteristics (also see [36,37]) and that in these cases the CoP theory is applicable for analytical purposes.

## Methods

### Setting

To explore the interaction between primary care CoPs and a service improvement programme, this study was conducted within the Collaboration for Leadership in Applied Health Research and Care for Greater Manchester (GM CLAHRC). CLAHRCs are five-year collaborative partnerships established in 2008 between universities and NHS organisations, aiming to create innovative ways of producing and implementing applied health research by bringing together producers and users of research [38]. By performing the three interlinked functions of conducting high-quality applied health research, implementing research findings in clinical practice and increasing the capacity of NHS organisations to engage with and apply research, the CLAHRCs are seen as a way of addressing the second translational gap, i.e. a gap in the translation of new medical interventions into everyday practice [39]. Being co-funded by the National Institute of Health Research (NIHR) and local NHS trusts, the CLAHRCs are encouraged to develop a collaborative model of ownership, with a range of stakeholders having vested interests in determining their agendas and tailoring the conduct of research to the specific needs of a particular region [40,41].

The study specifically looked at the Chronic Kidney Disease (CKD) theme of the GM CLAHRC Implementation Strand which worked with 19 self-selected general practices across Greater Manchester in 2009–2010. The theme aimed to increase the identification of patients with CKD in primary care and improve their management and treatment in line with existing scientific evidence. The CKD project utilised the Institute for Healthcare Improvement Collaborative model [42], which implies ‘participation of a number of multiprofessional teams with a commitment to improving services within a specific subject area and to sharing with others how they made their improvements, each from an organisation which supports these aims’ [43]. Since knowledge sharing was supported both *within* and *across* multiprofessional teams, whose members voluntarily opted for participation, had similar interests in CKD and/or service improvement, and interacted on a regular basis, the implementation project was seen as a vehicle for the development of CoPs within and across general practices taking part in the collaborative [22].

Each participating general practice appointed an improvement team comprised of a GP, a practice manager and a practice nurse who were in charge of introducing change in their practices and who kept close contact with the external CLAHRC CKD facilitation team. This team, in turn, comprised two nephrologists, a management academic, a programme manager, a data analyst and two knowledge transfer associates (KTAs), all of whom interacted with the participating practices on a regular basis at quarterly learning sessions (with all improvement teams) and monthly meetings (with individual teams) as well as by phone or email if the need arose. Supplementing the collaborative approach, external facilitation drew on the experiences of the UK knowledge transfer partnerships and specifically focused on bridging boundaries between various stakeholder groups and enabling knowledge sharing between them [44]. The programme aimed to improve CKD related clinical procedures in participating practices, namely the way that CKD patients were diagnosed, coded and managed. It also involved wider administrative and communication aspects, such as performing a series of chronic disease register searches, modifying the procedures of biological sample collection, and engaging other clinical and non-clinical staff in the process of change [45].

### Data collection and analysis

An embedded case study design was chosen for this research project, with four general practices as individual subcases.^a^ The concept of replication logic [46] was deployed for purposeful sampling: two smaller general practices (Practice A and Practice B, with two GPs each) and two bigger practices (Practice C with four GPs and Practice D with six GPs) were recruited for the study. In light of existing empirical evidence showing that smaller primary healthcare teams tend to achieve higher levels of integration and participation than the larger ones [47], the sampling approach was based on an assumption that the practice size might be one of the factors influencing the dynamics of CoP formation and functioning within primary care organisations. Selecting two groups of practices with two practices in each group allowed both literal (within each group) and theoretical (across the two groups) replication [46]. In addition to purposeful sampling, recruiting these particular four cases out of 19 practices involved in the CKD project was also determined by the practices’ accessibility: at least three practice employees had to give their consent to participate in the study for the practice to be selected. In addition to the level of individual general practices, knowledge sharing across them was also explored, thus adding one more, supra-organisational, level of analysis.

As far as within-subcase sampling is concerned, the participants were recruited purposefully, based on their involvement in the CKD project and/or overall position within general practices. Semi-structured interviews were the main method of data collection which took place in 2010–2011. Twelve respondents (five doctors, three nurses and four managers/administrators) were recruited from the participating practices. Two practice managers employed by non-participating practices, two senior primary care trust (PCT) medical directors and four members of the CLAHRC facilitation team were also interviewed to collect additional information, of particular relevance at the supra-organisational level of analysis. In addition to semi-structured interviews, the study also included twenty hours of direct observation, conducted predominantly at learning sessions and practice meetings, and the analysis of relevant documents and artefacts produced by the facilitation team and participating general practices throughout the project. Interviews were tape-recorded and transcribed verbatim, hand-written field notes were digitalised, and all resulting text documents were subsequently coded with the aid of NVivo software.

The process of data analysis was organised in three rounds. In the first round, template analysis [48] was used to organise emerging codes into a number of categories (e.g. boundary, identity, boundary objects, boundary encounters, etc.) which were informed by the CoP literature and reflected the structure of the interview guide. In the second round of data analysis, sorting data by codes and categories gave way to summarising and synthesising data within each of the four subcases, with a special focus on professional boundaries within improvement teams and intra-organisational boundaries between the teams and the rest of an organisation. The third round of analysis examined inter-organisational boundaries and also explored the relationship between knowledge sharing and different types of boundaries across the four subcases. This was assisted by matrix analysis [49], which was deployed for comparing and contrasting the data obtained from four participating general practices as well as from different research methods (interviews, observation and documentary analysis), thus serving the purposes of triangulation. (See Additional file 1 as an example of cross-subcase and cross-method triangulation). In addition to triangulation, research findings were also validated by peer-debriefing between the three co-authors as well as by member checking with research participants [50]. Research participants were offered an opportunity to provide feedback and comments on the transcripts of their interviews and on an earlier version of this paper. They were asked if the interpretation of data made sense, whether it was supported by sufficient evidence and whether the overall account was realistic and accurate. Feedback received throughout this process was incorporated in the final version of the manuscript.

To explore the impact of knowledge boundaries on the implementation of the programme and emergence of multiprofessional and multi-organisational CoPs at different levels, the findings of the study are presented in the following way. The first subsection looks at the interactions within improvement teams which were driving change in their settings. Then the challenges to knowledge sharing between improvement teams and other intra-organisational groups are examined. Finally, knowledge sharing between improvement teams representing different primary care settings is described, with a wider exploration of the issues related to inter-organisational knowledge exchange in primary care. These issues are further elaborated in the Discussion, in which the findings related to professional and organisational boundaries are interpreted in light of the CoP theory, with a specific focus on the role of (both existing and emerging) CoPs in the implementation of service improvement initiatives.

## Findings

### Multiprofessional improvement teams

Implementation of CKD work in the practices was directed by improvement teams, each of them comprising a GP, a nurse and a practice manager. The roles, degrees of involvement and contributions of each of the team members differed across settings. In three cases (A, B and D), a GP provided overall leadership for the project; in Practice C, the nurse was the main driving force (Table 1). The multiprofessional nature of the improvement team in combination with a clear distribution of roles supported a focus on both the clinical and administrative aspects of the project:

I was part of a team that worked very well together, that worked closely together… The practice manager would run the lists off… she was responsible for collating lists and computer searches, because that is her role, and she does that very well. She would then hand them to the GP, who did her role in that. She would correctly Read Code people, look if they were people that she already knew about, people that we already had in hand and we were going to do a repeat renal profile in the three months. And then she would hand that to me and I would play my part, which was sending letters out, getting the patients in to see me, collecting the urines, doing the blood pressures, making sure that we had the blood tests done. And we worked well together as a team. We all knew our roles… Teamwork was second to none. (PN1)^b^

**Table 1:** Characteristics of the subcases

	**Practice A**	**Practice B**	**Practice C**	**Practice D**
**Number of GPs**	2	2	4	6
**Who provided leadership in the improvement team**	Dispersed leadership with the GP as the main coordinator		Practice nurse	GP
**Knowledge sharing boundaries encountered by the team**	Between the improvement team and the receptionist staff		1) Between the improvement team and the receptionist staff	
			2) Between the improvement team and the rest of the clinical staff	

Motivations to join the collaborative differed across the members of the improvement team, the most frequently reported ones including an interest in CKD (more typical for GPs and nurses), a need to improve the CKD register (more typical for practice managers), a general passion for learning new things and improving patient care, and a possibility to gain financial benefits for the practice through getting extra QOF^c^ points. Regardless of their initial motivations, many interviewees were very enthusiastic about the CKD work they were doing:

I live, breathe and sleep CKD. My husband is sick of hearing about CKD and it’s all I talk about, so I can’t really be any more committed or interested than I am. (PN2)

I think the three of us have the motivation, because we had an interest in it and we wanted to get it right. We wanted it to work. (PM4)

Multiprofessional differences between the team members were not perceived as a barrier to the implementation of the project, this view being shared both by the practice staff and by external facilitators:

I don’t think any problems that we encounter are as a consequence of the different roles of the practice manager, the nurse, or the GP, because they’re all working for the same purpose… I find in general, I think, that most people in a practice are working toward the same thing, because they have a common practice goal, but they also have individual professional goals. But they’re all largely co-dependent on the same outcomes. (EF1)

…Here we work so much together on everything else because we are a practice. So, we have to share knowledge on everything else. So, doing it with the CKD was nothing different to how we would generally. We were used to sharing that knowledge. We were used to interacting: the nurse with the doctors, the nurse with me. So, it didn’t cause any major disabilities that way because that’s how we work anyway, you know, we share. (PM6)

As could be inferred from the last quote, successful communication and coordination in the improvement teams were largely predetermined by the history of previous relationships in the practices, especially in view of the fact that in many cases GPs, nurses and practice managers had spent years working together in the same practice. However, participation in the collaborative was perceived by many interviewees as further improving these processes, especially in Practices B and C, where some of the improvement team members were relatively new to their practice roles:

I would say it brought us together as a team better, and it helped I think us find out areas within the team, really, how we can work within that team, and our strengths and weaknesses, within those who were doing the collaborative, but also the actual practice itself: it certainly improved our communication… So I think it’s been teamwork, communication, and realisation that we really want to improve the practice and we can do it. (PN3)

The majority of interviewees repeatedly emphasised their commitment to the general practices they were working in. When asked whether their profession or organisation was the main locus of identification for them, they either tended to prioritise their organisational membership over professional affiliations or argued that the two are complementary and cannot be viewed as separate entities:

I feel as though being here, I’ve been able to sort of develop more, because I’ve been valued more, and I’ve had to do more… So I feel as though they’re encouraging me to be the nurse I want to be. (PN3)

### Getting the rest of the practice on board

The dynamics of the interaction between the improvement team and the rest of the practice differed depending on the practice size and number of GPs employed (Table 1). In smaller practices (A and B), CKD related issues were routinely discussed at weekly practice meetings involving all clinicians and a practice manager. Monthly improvement team meetings, which were run by an external facilitator, were usually attended by other GPs and nurses, including those who were not formally part of the improvement team. Involvement of all clinicians working in the practice in these interactions enabled direct knowledge exchange about the CKD project, facilitated incorporation of newly introduced approaches to the identification and management of CKD patients in practice routines, and provided senior support perceived as crucial for achieving sustained improvement in the practice:

So I think that helped as well, that [the senior partner] came to the meetings with [the external facilitator]. So she was instrumental… sort of a side with the improvement team. She was like an extra member. (PM1)

…I wanted to learn as well, along with the others, to see what’s going on. And if I didn’t know anything about CKD and if the patient comes to me, what do I do next? I didn’t want to present a blank face to the patients. Because being a small practice, we can all get involved, which probably is not possible in a big practice. (GP4)

In larger practices (C and D), the work of the improvement team did not seem to be so well integrated in the functioning of the whole practice. The improvement team members had their own regular meetings and the main challenge they had to overcome was getting the rest of the clinical staff on board, which meant explaining the importance of the CKD work for both patient benefit and financial outcomes, sharing knowledge about the identification and management of CKD patients and making sure that the changes introduced by the project became embedded in day-to-day clinical practice. This was mainly achieved by communicating to the practice staff at various types of practice meetings as well as during informal exchanges between colleagues:

The GPs have meetings every Tuesday. The CKD is actually the main thing on the agenda. They usually talk about that first before they talk about anything else. The practice nurse is coming to that meeting once a month, and we do have education meetings for all disease areas regularly as well, and even the receptionists are included in what we’re doing, so it’s not just a nurse or a GP thing. It’s the whole practice that are involved in what goes on here. (PN2)

…There are informal meetings like you’re standing around some place and start talking about an issue or something… (GP3)

Despite the fact that CKD was discussed at practice meetings and all staff members received relevant education from the improvement team members, both of the large practices experienced some problems in getting the rest of the clinical staff to become more involved in the project or change the way they manage CKD patients. In Practice D, all GPs had their own clinical areas of interest, whereas Practice C employed two part-time GP locums who were seen (and actually saw themselves) as less committed to the surgery and were not involved in any administrative work. In both practices, those GPs did not seem to identify themselves with the work performed around CKD by the improvement team. Knowledge and practice developing around the CKD improvement project, although not actively resisted by other clinicians, were seen by the latter as the prerogative and responsibility of the improvement team:

…What happens is if you take up a thing, people tend to load all the results and everything onto you to take a decision about the patient… Not everyone was entirely keen, in the sense that they had lots of other things on their plate, with the QOF and other things, so they were more concentrating on other things. (GP3)

Two more challenges to the implementation of the CKD work were experienced by all practices taking part in the study. The first of them was related to involving the practice receptionist staff in the work and making sure that they followed the new procedures introduced in the practice. This challenge was identified and addressed quite early on through involving the admin team in the meetings, explaining to them the reasons behind new arrangements and presenting the procedures they were required to follow in a simple, clear form. Another challenge included allocating protected time for CKD work which was being implemented on top of the routine clinical and administrative work in the practice. This issue was addressed by the collaborative facilitation team who had resources to cover the additional costs of buying out clinicians’ time and thus enabling the surgeries to hire locums to backfill the time that was spent by the members of the improvement teams on CKD work. It was generally felt that without these financial resources the participation of the practices in the CKD collaborative would have been less likely. Additional resources also helped to overcome resistance of those GPs who saw the CKD work as peripheral and were worried that CKD would take priority over other clinical areas and the latter would suffer.

### Inter-organisational collaboration in primary care

Throughout the course of the programme, it became clear that the level of communication between the practices taking part in the project was low, which manifested itself in several ways. Knowledge sharing between the practices, all of which were supposed to learn from each other about the identification of CKD patients, was limited to the quarterly learning sessions led and facilitated by the collaborative facilitation team. WebEx teleconferences organised in order to facilitate knowledge exchange at a distance attracted a limited number of usual suspects—the most enthusiastic and pro-active participants, and failed to secure a wider participation. An online community launched at the collaborative website for the same purpose was never used by the members of the improvement teams:

…Aside from the learning sessions, people didn’t really speak with each other. We didn’t get the forum community going on the web that we thought we might do, and discussion threads going on so that people could do a lot more inter-PCT sharing of learning. (EF3)

Lack of communication between general practices was acknowledged as a challenge by PCT managers, improvement team members and the collaborative facilitation team. It was generally felt that the current level of knowledge sharing between primary care organisations is not sufficient for standardisation of care, spread of best practice and developing a shared strategy to address the current reform of primary care. The insufficiency of existing mechanisms of inter-organisational collaboration was, at least partly, compensated for by the availability of external facilitators who played the role of knowledge brokers transferring contextualised project-related knowledge from one practice to another:

It would be easier for the spread if there was better communication between them… If we’d have known that they were talking to each other, we would do less of the putting in of the KTAs^d^ to actually do that role; because the KTAs, that middle person, it’s almost like they’re the hub, who’s going out and sharing learning between all the five practices in that PCT. “Practice X is doing this, so you could do this.” “That’s worked really well in practice Y; I think that would work in your context. Let’s try that.” So you would cut out that middle person, and almost… the KTA as that change agent isn’t going to be around forever. They’re only going to be around for the duration of the project. So if you could get people working more together, you wouldn’t need that facilitation and support, because they’d be doing it themselves. (EF3)

Interviewees suggested a number of explanations as to why knowledge sharing between practices remained limited. First, the practices saw themselves as independent individual businesses competing for position in a PCT league table, for having ‘the best registers’ and hence higher QOF points, and for attracting more patients, if the practices’ catchment areas overlapped. Business-style competitive rivalry was potentiated by the ethos of confidentiality and strong organisational identification:

…I think we’re very protective of what we’ve got, and I think that will always be a barrier because it’s always been. When I started working in primary care in ‘97, it was them and us. We didn’t share any information at all. It’s calming down now and it is getting better, but I think the only barrier will be “I don’t want them to know what we did well and them doing it and them being better than us”. (PM6)

Second, while the formal channels of inter-organisational communication (such as educational events for GPs, practice managers’ forums and nurses’ forums) provided some opportunities for knowledge exchange, they were predominantly organised along traditional uniprofessional lines, focused on didactic education rather than interactive discussions, and were not always well attended. The development of these communication channels markedly differed across different PCT areas, in some of which practice managers’ forums, for instance, were perceived as less useful than in others:

…We don’t really share good ideas. It’s not shared, even though we have practice managers’ groups; and Health Authority, they have a practice managers’ group where they sit in, and we have one outside the PCT to try and encourage other practice managers to come in and talk about what they know, what they specialise, and all the rest of it. But it doesn’t actually work, because people hold back on what they’re actually doing. (PM5)

It should be noted, however, that the majority of respondents agreed that inter-organisational collaboration in primary care has improved over the last five years; this development, however, appeared unequal across different PCTs. Progress achieved in more ‘collaborative’ areas was attributed to the increasing use of email communication; publicising open comparison data by PCTs (although this was seen by people from less collaborative areas as a barrier to collaboration); and, most importantly, previous involvement of pro-active practices in practice-based commissioning (PBC).^e^ GPs and practice managers involved in PBC reported sharing protocols, working together on redesigning referral pathways and collectively discussing commissioning arrangements, but many of them still perceived inter-organisational collaboration as difficult.

## Discussion

### Professional boundaries

Our findings show that professional boundaries were not perceived by respondents as a barrier to knowledge sharing and implementation. This could be explained by a number of factors. First, interprofessional interaction between the members of the improvement team did not challenge the existing power structures within their organisations, with GP partners providing clinical leadership and retaining final authority in terms of clinical and administrative decision-making throughout the project [51]. At the same time, substantial autonomy was granted to the subordinate members of the team, who were in some cases allowed to drive the project and determine the overall approach to implementing change. Second, the CKD project (similar to many other activities undertaken by the primary care staff) included a combination of managerial, clinical, technical and other aspects, addressing which required a genuinely multidisciplinary approach. Finally, and perhaps most importantly, in most cases the members of the improvement teams had worked closely together in the same practice for at least several years—in other words, the relationships determining the improvement team dynamics had existed prior to the start of the programme. Effective communication within the teams was therefore largely determined by the processes taking place in wider multiprofessional CoPs existing prior to the launch of the CKD programme, although participation in it improved communication and teamwork even further.

Similar to observations made by Gabbay and le May [15], this study has shown that despite the presence of inherent epistemic differences stemming from their different roles and professional backgrounds, GPs, practice nurses and practice managers working in the same practice may develop a level of knowledge sharing and collaboration that corresponds to a multiprofessional CoP. Division of labour in these CoPs provides differentiation rather than fragmentation and does not preclude the formation of a shared domain of knowledge and practice which is enabled by close operational proximity and sharing common values [52]. It could be assumed that the formation of such multiprofessional CoPs is more likely within primary care than in secondary care based multidisciplinary teams because the latter operate in far more complex and hierarchical organisations, are explicitly focused on clinical decision-making traditionally seen as a jurisdiction of the medical profession, and may be misused as a way of privileging the knowledge of more powerful team members and legitimising these (in fact unidisciplinary) decisions by deploying a multidisciplinary discourse [27]. It should also be noted that in order to respond to growing pressures to manage workload more efficiently, GP partners have to delegate some of their clinical and administrative duties to practice nurses and practice managers, which gives these (traditionally subordinate) groups more autonomy and power in relation to those at the bottom of the hierarchy (e.g. healthcare assistants, receptionist staff), but at the same time does not significantly violate GPs’ professional dominance and power [51,53]. A combination of these factors increases the level of interdependence and widens the domain of shared practice that may form the basis of a multiprofessional CoP, which does not need to be an egalitarian structure void of internal power relations [9,13].

### Intra- and inter-organisational boundaries

The absence of major interprofessional tensions within the improvement teams did not mean that the sharing of CKD related ‘knowledge-in-practice-in-context’ [15] met no boundaries within the participating general practices. One of these boundaries lay between the improvement team (or, as was the case in smaller practices, between a wider CoP including all clinical staff plus a practice manager), on the one hand, and the receptionist staff, on the other. This boundary was mainly syntactic (the receptionists did not understand the professional jargon) and was successfully bridged by ‘translating’ the message into the lay language. Another boundary, typical for larger practices with a higher level of GP specialisation and a higher proportion of part-time GPs, lay between the improvement teams and other clinical staff, many of whom were members of other subgroups centred on their own areas of clinical interest, such as chronic obstructive pulmonary disease, diabetes or gynaecology. This boundary was mainly semantic: some clinicians did not share the same meanings about the importance of the CKD work as the members of the improvement team, did not seem to sufficiently identify themselves with the CKD project and failed to internalise the new arrangements in their ‘clinical mindlines’ [15]. It also reflected the influence the CKD work had in an organisation as well as the fit between the project and the interests of individuals and groups involved. Intra-organisational boundaries did not influence the immediate outcomes of the project as the improvement team members were doing all the necessary work, but were recognised as a potential challenge for long-term sustainability of change in the practice.

At the supra-organisational level, the formation of a functional CoP that would include all improvement teams participating in the collaborative was perceived as problematic due to the presence of strong pre-existing organisational boundaries between general practices. This reflected an acknowledged problem of insufficient communication between general practices underpinned by the business nature of primary care organisations, competition between them, strong organisational identification, as well as looseness and unequal development of inter-organisational networks, such as locality meetings or practice managers’ forums. In some areas, a certain level of inter-organisational knowledge sharing was achieved between practices which shared a history of participation in long-term initiatives, such as PBC, but this only involved a relatively small number of pro-active GPs and practice managers. Overall, the role of communities and networks cutting across organisational boundaries in sharing CKD related knowledge was insignificant, with practitioners’organisations being the primary locus of work identification. All of these factors resulted in the formation of strong inter-organisational boundaries which significantly limited knowledge sharing between the participating practices and were partially bridged by external facilitators performing a knowledge brokering function. Given the presence of a strong organisational identification and the interconnected, overlapping nature of the CoPs operating within general practices, it is possible to view primary care organisations as constellations of CoPs united by a shared organisational culture and identity. As a result, constituent (multiprofessional) intra-organisational CoPs tend to become tighter than those (predominantly uniprofessional) networks of practice that cut across organisational boundaries in primary care.

### Existing and emerging CoPs

Combining the findings of this study with the results obtained by previous research [15], it could be argued that the CoP landscape in primary care is complex and that its analysis cannot be reduced to a simple interaction between various uniprofessional CoPs co-located in a organisational setting. The implementation of the CKD project has demonstrated that this landscape includes a number of overlapping uniprofessional and multiprofessional communities and networks of practice, some of which are confined to a given primary care setting and some cutting across organisational boundaries (Table 2). Configuration of boundaries in this landscape significantly influenced the processes of project related knowledge sharing, shaped the implementation of the project and largely determined the challenges faced by the initiative in terms of spread and sustainability of change. It should be emphasised that the majority of knowledge sharing related to the CKD project was occurring within improvement teams or wider multiprofessional CoPs contained in general practices, with some information circulating through traditional uniprofessional routes of communication (i.e. among doctors). Knowledge sharing along these uniprofessional routes did not always lead to a change of clinical practice due to the presence of an identity boundary, whereby some GPs were not interested in CKD because they had a different area of specialisation and some were locums with a lower degree of organisational identification. This reinforces the findings of previous research showing that the growing specialisation of GPs leads to the transformation of their traditional ‘biographical’ identity into a ‘consultant’ or ‘specialist’ identity [51] and may potentially threaten the coherence of their shared professional identity [54] thus impeding knowledge sharing.

**Table 2:** Landscape of communities of practice within and across GP surgeries

	**Multiprofessional**	**Uniprofessional**
**Within primary care organisations**	Groups centred on an area of interest (e.g. CKD improvement teams)	GPs working in the same practice
	Clinicians and the practice manager	Receptionist staff
**Across primary care organisations**	PBC groups of GPs and practice managers	GPs’ informal networks
		Practice nurses from the same geographical area
		Practice managers from the same geographical area

In Practices A and B, multiprofessional improvement teams could be viewed as subgroups within wider organic CoPs operating within these practices (and, in turn, composed of the clinical staff and the practice manager), whereas in larger primary care settings (e.g. Practice D) these teams may resemble small CoPs in their own right. It could thus be assumed that the formation of new CoPs within primary care improvement initiatives is likely to be confined to individual organisations, contingent on pre-existing relationships, and enabled by a shared history of working, learning and sense-making over a relatively long period of time, rather than by the introduction of an external, time-limited change initiative. The service improvement initiative described in this paper was deliberately situated in the context of existing multiprofessional CoPs and managed to successfully utilise them, exercising a certain degree of influence on the internal processes in these CoPs through context-sensitive facilitation. At the same time, creating a new CoP bringing together all practices taking part in the initiative was less successful. All participants were interested in CKD and quality improvement; the practices were recruited on a voluntary basis and their participation was incentivised; external facilitators specifically addressed the issue of inter-organisational collaboration by providing a forum for both face-to-face and online communication—all these factors did not, however, lead to the formation of a functional multi-organisational CoP centred on the initiative. The development of such a CoP in the CKD collaborative was hampered by strong organisational boundaries, lack of time and resources for inter-organisational collaboration, and de-prioritisation of inter-organisational knowledge sharing which was not seen as important for achieving individual organisational aims. This study, therefore, concurs with the findings of previous research showing that functional multi-organisational CoPs in the NHS collaboratives might fail to develop [22] but highlights the role of pre-existing boundaries, rather than the features of the collaborative approach, as a major obstacle to their formation.

### CoPs and service improvement in primary care

The findings of this study make us question whether deliberate CoP engineering advocated by the instrumental perspective on CoPs could be considered the most appropriate knowledge utilisation tool in the context of primary care service improvement in general and quality improvement collaboratives in particular. Manipulated emergence of CoPs *de novo* is likely to be problematic, with structures that emerge in this process requiring investment of substantial resources and yet remaining unsustainable. At the same time, the analytical perspective on CoPs, aiming to study issues related to their boundaries and identities, offers little more than passive analysis and observation, giving no prescriptions as to how existing CoPs could be utilised in the process of service improvement. Consequently, this paper suggests a *developmental approach* to CoPs, which lies midway between the analytical and instrumental perspectives (see Table 3) and calls for the maximal utilisation of existing organic CoPs and improving communication within and between them rather than attempting to construct a heterogeneous CoP centred on a time-limited improvement initiative. This approach includes mapping existing CoP landscapes that are relevant for a given service improvement initiative, analysing the configuration of boundaries, roles and identities in these landscapes, and combining external and internal facilitation to make the boundaries between all CoPs involved more permeable and enable the incremental development of these CoPs through participation in the initiative. This may include expanding CoP membership by attracting new members, widening its scope of interests and increasing awareness of the internal CoP dynamics.

**Table 3:** Three perspectives on CoPs and their potential application in healthcare service improvement

	**Analytical perspective**	**Instrumental perspective**	**Developmental perspective**
**Purpose**	A theoretical heuristic to analyse practice, meaning, identity and learning	A knowledge management tool aiming to deliberately engineer, or ‘cultivate’ CoPs	Analysis of relevant CoPs and their characteristics accompanied by the facilitation of their development
**Types of CoPs prioritised**	Existing or naturally emerging, organic, often uniprofessional	Deliberate, often multiprofessional and/or multi-organisational	Multiple, overlapping CoPs forming wider landscapes of practice
**Potential application**	Researching boundaries, identities and their influence on knowledge sharing; informing theory-driven implementation interventions [17]	Delivering joint projects by CoPs comprised of committed and legitimate members, placed in favourable context and supported by infrastructure and resources [55]	Implementing service improvement interventions in complex multiprofessional and multi-organisational contexts with numerous barriers to knowledge sharing

A number of important issues have to be taken into account when utilising the developmental approach to CoPs in the primary care context. First, previous literature has shown that organic CoPs are likely to be resistant to external managerial attempts to drive them in a given direction [56]. This, therefore, rules out a directive, top-down approach to service improvement, underscores the importance of co-production and shared ownership of the initiative between the local CoPs and external facilitators, and requires a nuanced and facilitative approach to implementation. Second, the complex, overlapping, multi-level nature of the CoP landscape in primary care, as well as the multiplicity of communication channels within and between CoPs, underscores the need for facilitators to identify and target actors with simultaneous membership in a number of intra- and extra-organisational CoPs to secure their early involvement in service improvement and designing the strategy for its spread and sustainability. Not only will these internal knowledge brokers have to be perceived as legitimate, competent and non-threatening by participating organisations; they will also need to possess knowledge and skills related to dealing with intra- and inter-organisational boundaries described in this paper, which goes beyond a more traditional approach to quality improvement focused on data, tools and targets. Third, it should be remembered that knowledge co-created by a CoP might become sticky at its boundaries, which needs to be counter-balanced in order to achieve long-term sustainability of change and its embeddedness in organisational routines. Finally, if the improvement programme is meant to involve inter-organisational collaboration and allow the spread of best practice across individual organisations, a strategy for bridging organisational boundaries should be planned well in advance, with maximal utilisation of existing inter-organisational networks and channels of communication.

### Limitations

This study is not without limitations. First, it has only looked at general practices which voluntarily opted for participation in the project, were enthusiastic about it and could be classified as innovative organisations. It is known that primary care teams open to innovation and change are more likely to work well as a team [57,58], which could explain the development of functional multiprofessional CoPs in the context of the study. It may, however, be assumed that less innovative practices might have a different intra-organisational dynamic. Second, a relatively small sample size can also be considered a limitation; this was counterbalanced by corroborating the findings emerging from the participating practices by the information collected from PCT representatives and external facilitators who had access to a larger number of primary care settings. Third, the volume of raw data produced by interviews exceeded the volume of data collected by direct observation and documentary analysis. This inequality is reflected in the Findings section, which has relied on interview quotes, rather than excerpts from field notes or documents, to illustrate the key findings. However, as shown in Additional file 1, observation and documentary analysis were used to validate the findings obtained by interviews as the main method of data collection. Finally, since the implementation of the project only involved the ‘core’ practice staff, the paper has chosen not to look at the communication, boundaries and collaboration between general practices and community-based representatives of broader primary care multidisciplinary teams, such as district nurses, community matrons and health visitors. The views of the receptionist staff and features of their CoPs might also be an interesting area for future research.

## Conclusions

This paper has explored the landscape of interconnected CoPs influencing the implementation of an improvement initiative within and across primary care settings. This landscape of practice has a number of specific features. First, multiprofessional CoPs acting within individual practices are instrumental for the sharing of knowledge produced by the improvement initiative and, in spite of inherent differences in their members’ knowledge base and status, can successfully bridge professional boundaries and achieve a sufficient level of internal integration without major tensions or conflict. Second, although knowledge circulates relatively easily within such a CoP, barriers to knowledge sharing might emerge at the boundary separating it from other groups existing in the same organisation. These barriers are often underpinned by variability in the degree of identification with the initiative and the organisation as a whole rather than by interprofessional differences. Tending to be more pronounced in larger general practices, such intra-organisational boundaries may threaten the sustainability of improvement initiatives. Finally, although some uniprofessional communities and networks cut across organisational boundaries, they make the latter only partially permeable for knowledge flows. Inter-organisational knowledge sharing and collaboration may remain problematic and unequally developed across different areas due to historical factors, competition and strong identity boundaries between individual general practices, which may present an obstacle to the spread of best practice.

Even with relatively permeable professional boundaries, the engineering of functional multiprofessional CoPs in primary care is likely to be contingent on the relationships between professional groups existing prior to the launch of the initiative and might still require an investment of substantial resources to incentivise participation and facilitate CoP functioning. Manipulated emergence of a multi-organisational CoP that brings together representatives of all practices taking part in a change initiative is even more problematic due to strong organisational boundaries; overcoming such boundaries would take more time and effort than is available in a typical service improvement project. In light of these findings, this paper argues for a developmental approach to CoPs which builds on the strengths of the analytical and instrumental perspectives described in the CoP literature but avoids the extremes of passive observation and deliberate construction of CoPs. This pragmatic, situational approach combines a reflexive analysis of boundaries, membership and dynamics in the existing CoP landscapes with the facilitation of CoPs’ internal development and potential modification of these CoPs targeting both intra-organisational and inter-organisational boundaries. By doing so, it offers a potential to enhance the spread and sustainability of service improvement and improve the permeability of boundaries to knowledge flows without radically reconfiguring organic landscapes of practice operating in the field.

## Endnotes

^a^The study was reviewed and approved by the North West 8 Research Ethics Committee—Greater Manchester East. ^b^The following abbreviations are used to indicate respondents who are quoted in this section: PN—practice nurse; GP—general practitioner; PM—practice manager; EF—external facilitator. ^c^The Quality and Outcomes Framework (QOF) is a voluntary annual reward and incentive programme for all GP surgeries in England, detailing practice achievement results. ^d^Knowledge Transfer Associates—members of the CLAHRC facilitation team working with the practices. ^e^Practice-based commissioning (PBC) is a UK Department of Health initiative designed to give primary care professionals the power to decide how NHS money is spent in their local area.

## Competing interests

RK was a recipient of the CLAHRC PhD studentship, KW is Associate Director of the NIHR Health Services and Delivery Research Programme (HS&DR) and GH is an Academic Lead for the NIHR CLAHRC for Greater Manchester, but they write here in a personal capacity. The views and opinions expressed therein are those of the authors and do not necessarily reflect those of the NIHR HS&DR or the NIHR CLAHRC for Greater Manchester.

## Authors' contributions

RK reviewed the literature, conducted the fieldwork and drafted the manuscript. KW and GH revised the manuscript. All authors read and approved the final manuscript.

## Supplementary Material

Additional file 1 Cross-site and cross-method triangulation of research findings. Click here for file
