# Supplementary material for: Managing boundaries in primary care service improvement: A developmental approach to communities of practice
Source: Implement Sci. 2012 Oct 15;7:97. doi: 10.1186/1748-5908-7-97 (PMC3514317; doi:10.1186/1748-5908-7-97)
Supplement: Additional file 1 — Cross-site and cross-method triangulation of research findings. [file 1748-5908-7-97-S1.doc]

**Additional File 1.** Cross-site and cross-method triangulation of research findings.

|  | **Practice A** | | **Practice B** | | **Practice C** | | **Practice D** | |
| --- | --- | --- | --- | --- | --- | --- | --- | --- |
|  | ***Face-to-face interviews*** | ***Observation & Documents*** | ***Face-to-face interviews*** | ***Observation & Documents*** | ***Face-to-face interviews*** | ***Observation & Documents*** | ***Face-to-face interviews*** | ***Observation & Documents*** |
| **Interaction within multi-professional teams** | ‘…It’s something with being a small practice: we’ve always worked well as a team in getting things done. I think it’s been just another team experience, really, that’s highlighted how well things can work when everyone pulls together.’ (GP5) | The meeting takes place in the lobby of the general practice. The team seem to be very happy to see each other and the change agent. The atmosphere is friendly, and all members of the improvement team equally participate in the discussion. | ‘I don’t know: it’s just interaction of different personalities, and we’ve all got on very well, but is that because… I don’t know… we’re similar personalities, we want the practice to improve… people are committed…’ (PN3) | The change agent (EF2) asks a question or gives a (general) suggestion - then the improvement team start discussing it linking it to the practicalities and the context of their practice. The discussion is very hands-on, very practical, and everyone seems to be actively involved. | ‘I think we’re a team, and I think we work well as a team, and there’s a good skill mix, and everyone has their own roles and responsibilities, and they might kind of interlink into someone else’s role. But I think it works well.’ (PN1) | All questions raised by the change agent get quickly answered: the practice manager deals with administrative issues while the nurse controls the process of the clinical follow up. Lead GP is not present (he is a part-timer and is only in the practice for two days a week). | ‘…We had no problem at all really. There is a lot of respect of each other. We know we couldn’t have done this without each other’s help. So, there is respect.’ (GP1) | Practice nurse, practice manager and lead GP are equally involved in the discussion. A lot of questions about PBC are addressed by the practice manager who sits on the PBC consortium. The atmosphere is friendly; all issues get discussed very quickly and productively. |
| **Involvement of other clinicians in the Chronic Kidney Disease project** | ‘[Senior GP] was open for anything that’s going to improve practice and also improve prevalence and improve understanding. So she was quite open to that and quite open to periodic meetings to keep her up to date. So that wasn’t a problem in this particular practice.’ (GP5) | The senior GP (GP4), who is not formally part of the improvement team, is present at the improvement team meeting. It looks like she is familiar with all the work performed within the project. She takes part in discussing the results of the first phase of the Collaborative and gives the change agent (EF1) advice about recruiting potential participants for the next phase. | ‘As far as the clinicians went, we went through the NICE guidelines for CKD… We made sure that everyone was aware of the full clinical situation of what to do with CKD… And we created kind of guidelines of what we have within the practice… And we got the trainees involved, as well, and they took kind of a role in managing these people, as well. (GP2) | Senior partner, who is not formally part of the improvement team, is also present in the meeting. She seems very enthusiastic about the work being done.  A powerpoint presentation on managing the CKD patients was prepared by one of the GP trainees (a copy obtained from the practice manager). | ‘The doctors seemed… the difficulties we found here was… because we were so focused on doing the CKD, the doctors got quite worried that we weren’t focusing attention on the other registers as well. So, CKD was kind of taking priority over everything else, what we need to do for all of our other patients. That was the major difficulty at the time…’ (PM6) | In the meeting, the team members discuss one of the doctors, ‘who is not very familiar with the CKD protocol’. It is also mentioned a couple of times that a senior clinician is not very interested in the CKD work as ‘he is busy doing other things’.  My impression is that there is a *power differential* between the senior GP and all other employees (including other doctors)—does not look like a professional boundary between groups. | ‘…You need somebody that will drive this. You have to be constantly on the back of colleagues, GPs as well… You think they have the knowledge, you think they have and yet, two months down the line you see gross mistakes as to the way that they have put them on the register or omitted them completely, or the management treatment that you have spoken through and you think they have understood.’ (GP1) | Only the members of the improvement team are present in the meeting. None of other GPs are there. The team discuss how to educate new staff members, acknowledging that the sustainability of change and its spread beyond the improvement team is potentially problematic. Lead GP (GP1) and practice manager (PM4) are also concerned whether the CKD work will continue in the practice after the lead GP retires. |
| **Involvement of receptionists** | ‘So I think we just worked really well together. It might have been in a way advisable to possibly have a member of the Reception team involved on the improvement team. I’m not sure. I haven’t made my mind up about that.’ (PM1) | No reception staff were present at the improvement team meeting. | ‘…We realised… that we weren’t involving the reception staff with just general, the run-of-the-mill work, really; we felt as if though needed to have just general meetings with the reception staff, so that was a forum for when we can discuss with them about the Collaborative.’ (PN3) | No reception staff were present at the improvement team meeting.  Context assessment conducted by the facilitation team in this practice showed that the staff ‘were willing to try new approaches for service improvement but also that the environment is not always conducive to the sharing of ideas and that there is not always sufficient recognition for good work.’ | ‘…Whereas the receptionists, they can do their work within their hours, but I don’t think they get kind of rewarded… If they were developing a protocol, one of the receptionists, that she would know would get used by all the doctors and all the receptionists, they would probably put their hours in.’ (PM6) | No reception staff were present at the improvement team meeting. | ‘The receptionist staff didn’t know which colour top to send off the urine in; because of that, because of our weekly monitoring, our nurse actually devised a poster that we’ve now put in reception, with the colour, the actual pot, the colour of the pot top, and then which sample that goes in.’ (PM4) | No reception staff were present at the improvement team meeting.  The poster with clear and concise instructions about using different urine pots is put on the wall in the receptionists’ area. |
| **Change agents as boundary bridges** | ‘[Change agents had] knowledge in terms of if there was something we were stuck with they were able to go and find out, be that liaising with secondary care members, be it liaising with other practices in terms of, “If you’re finding this difficult, I’ll see what other practices are doing and see if I can come up with any ideas.”’ (GP5) | When discussing the problem of urine pot labelling at the practice meeting, the change agent (EF1) mentions that *in another practice,* there were cases when the pots were unlabeled and the lab reports were returned to the practice with no patient name. | ‘[The change agent’s role was] also to provide us with advice as to how to target certain groups, and *what other practices were doing.* (GP2) | In the practice meeting, the change agent (EF2) mentions the findings from a study (?) *from another PCT area* and suggests they might be useful for this practice. | ‘[The change agent] also was able to point out where other practices had been doing some work… So for instance… [he] said, “Well, practice A, they’ve actually looked at a particular medication in a particular age group,” And we hadn’t thought of that. So he was able to spread good practice.’(PM6) | In the practice meeting, the change agent (EF1) mentions having recently visited some other practices and says they seem to be experiencing a similar problem in the identification of the CKD patients. This is followed by a discussion about how the problem may be addressed. | [The change agent’s role was] also sharing good practice as to where he had been to see if that could be applied here. So, that was important; that was useful.’ (GP1) | No relevant evidence found in observation / documentary evidence. |
